# *SLC6A8* is a Potential Biomarker for Poor Prognosis in Lung Adenocarcinoma

**DOI:** 10.3389/fgene.2022.845373

**Published:** 2022-05-27

**Authors:** Yongfei Fan, Yong Zhou, Ming Lou, Zhaojia Gao, Xinwei Li, Kai Yuan

**Affiliations:** ^1^ Department of Thoracic Surgery, The Affiliated Changzhou No. 2 People’s Hospital of Nanjing Medical University, Changzhou, China; ^2^ Heart and Lung Disease Laboratory, The Affiliated Changzhou No. 2 People’s Hospital of Nanjing Medical University, Changzhou, China; ^3^ Department of Gastroenterology, Affiliated Cancer Hospital of Bengbu Medical College, Bengbu, China

**Keywords:** SLC6A8, lung adenocarcinoma, prognosis, biomarker, immunomodulator

## Abstract

**Background:** Recent studies have demonstrated that creatine can promote tumor metastasis and has implications for immune cell function. *SLC6A8* encodes a membrane protein that can transport creatine inside and outside the cell. However, there are currently no studies of *SLC6A8* in lung adenocarcinoma (LUAD).

**Methods:** In this study, the expression of *SLC6A8* in LUAD was analyzed using the Oncomine database, the Cancer Genome Atlas (TCGA) database, and immunohistochemical staining analysis. Survival analysis of patients with LUAD was performed using the cBioPortal and the Kaplan-Meier Plotter websites and clinical follow-up data. An analysis of the association between *SLC6A8* and the tumor immune microenvironment (TIME) of LUAD was performed through the TISIDB database and estimation of stromal and immune cells in malignant tumor tissues using expression data (ESTIMATE) algorithm. Then, based on the curated list of *SLC6A8*-related immunomodulators, three genes (*NT5E, CD40LG, CD80*) were selected to construct *SLC6A8*-related immune signatures to further evaluate the immune aspect of LUAD prognosis.

**Results:** Our studies indicated that *SLC6A8* was overexpressed in LUAD, and the high expression of *SLC6A8* was associated with poor survival. Genetic alteration of *SLC6A8* was also associated with a poorer prognosis. Furthermore, multivariate Cox analysis indicated that *SLC6A8* could be used as an independent risk prognostic factor. Then, immune infiltration analysis indicated that *SLC6A8* was also strongly associated with poor prognosis in the TIME of LUAD. A multivariate Cox proportional hazard model was then constructed, and was shown effective at identifying high-risk patients. Univariate and multivariate Cox analysis showed that the risk scoring of the model was an independent prognostic risk factor in LUAD.

**Conclusion:** *SLC6A8* may serve as a biomarker for poor prognosis in LUAD.

## Introduction

Lung cancer remains the world’s leading cancer-related cause of death (Siegel et al., 2021). Statistically, non-small cell lung cancer (NSCLC) accounts for 85% of newly diagnosed lung cancer, with lung adenocarcinoma (LUAD) being the most common subtype (Molina et al., 2008). Owing to the popularization of computed tomography (CT), some patients can be diagnosed early and as a result, the incidence and mortality rate of lung cancer has decreased (Oudkerk et al., 2021). However, the imaging specificity of CT is still poor, and even when nodules are confirmed, long-term follow-up is often advised instead of risking surgery (Cho et al., 2016; Lee et al., 2019). This leads to delays in treatment that may allow the cancer to develop. Therefore, it is essential to find biomarkers for early diagnosis and prognosis of lung cancer.

Tumorigenesis is dependent on the reprogramming of cellular metabolism as both direct and indirect consequences of oncogenic mutations (DeBerardinis et al., 2008; Loo et al., 2015; Pavlova and Thompson, 2016). A common feature of cancer cell metabolism is the ability to acquire nutrients from a nutrient-poor environment and to utilize these nutrients to both maintain viability and build new biomass (Loo et al., 2015). These nutrients are mainly glucose, fatty acids, and amino acids (Li and Zhang, 2016). The alterations in intracellular and extracellular metabolites that can accompany cancer-associated metabolic reprogramming have profound effects on gene expression, cellular differentiation, and the tumor microenvironment (Vander Heiden and DeBerardinis, 2017). One such metabolite is creatine, the metabolism of which has been recently shown to be associated with the progression of cancer. In hepatocellular carcinoma, creatine enters cells *via* cell membrane surface transport proteins to provide energy for metastatic survival (Loo et al., 2015). Creatine accumulation is also known to promote breast cancer cell survival and inhibit apoptosis by maintaining redox homeostasis in triple negative breast cancer (Li et al., 2021).

The creatine transporter solute carrier family 6 member 8 (*SLC6A8*) belongs to the subfamily of GABA transporters (GATs). This gene encodes a cell surface plasma membrane protein whose function is to transport creatine into and out of cells (Colas et al., 2020). Studies have shown that *SLC6A8* is not only associated with tumor development, but is also involved in the tumor immune microenvironment (TIME). The use of *SLC6A8* transporter inhibitors in colorectal cancer effectively inhibits creatine import, reduces intracellular phosphocreatine and adenosine triphosphate levels, and induces apoptosis in tumor cells (Kurth et al., 2021). In hepatocellular carcinoma, knockdown of *SLC6A8* significantly induced apoptosis and suppressed the migration and invasion of Hep3B and Huh-7 cells (Yuan et al., 2020). In recent studies, it was also revealed that *SLC6A8*-mediated creatine uptake and accumulation reprograms macrophage polarization by modulating cytokine responses such as IFN-g and IL-4, thereby altering macrophage-mediated immune responses *in vivo* (Ji et al., 2019), which could also be used to modulate the anti-tumor response of CD8 T^+^ cells by affecting creatine uptake (Di Biase et al., 2019). However, there are few reports on *SLC6A8* in lung cancer.

Given the associations between *SLC6A8* and various cancers, there is the possibility that it may serve as a biomarker. In this study, we analyzed the prognostic characteristics and immunological role of *SLC6A8* in LUAD. In doing so, we hope to identify whether it can be used as a tool for evaluating the prognosis of LUAD.

## Methods and Materials

### Acquisition of *SLC6A8* Expression Profiles

The expression of *SLC6A8* in pan-cancer was analyzed using the Oncomine database (https://www.oncomine.org/resource/login.html), the “Lung Cancer” section was selected for further analysis, followed by further filtering for its subtypes by datasets in the database. LUAD data (cancer = 535, normal = 59) was then downloaded from the Cancer Genome Atlas (TCGA) (https://www.cancer.gov/). We then conducted unpaired analysis of difference and paired analysis of difference for *SLC6A8* in normal and tumor tissues using the “limma” package in the R program (version 4.1.0). Unpaired difference analysis was used to compare *SCL6A8* expression levels in tumor tissues of 535 patients with paraneoplastic tissues of 59 patients, whereas paired difference analysis was used to compare *SCL6A8* expression levels in tumor tissues of 59 patients with paired paraneoplastic tissues of 59 patients.

### Construction of Tissue Microarrays

A tissue microarray (TMA) designated as TMA1 for the pre-experiment included 36 pairs of LUAD and paraneoplastic tissues from 16 female and 20 male patients. This was purchased from Superbiotek (Shanghai, China). The patients who contributed to TMA1 had an average age of 59.7 years [range: 34–81 years; stage: T1aN0M0 to T3N3M0 as per 2004 World Health Organization criteria (Travis et al., 2006)]. TMA1 was used to analyze paired differential analysis of *SLC6A8* expression levels in tumor tissues of 36 patients and paired paraneoplastic tissues of 36 patients. A second TMA, TMA2, was constructed with 51 LUAD tissues and 10 normal paraneoplastic tissues obtained from patients who underwent surgical resection in the Department of Thoracic Surgery of Zhongshan Hospital, Fudan University from January 2005 to December 2005. Ten paraneoplastic tissues were obtained from 51 LUAD patients randomly selected from 10 different LAUD patients. Complete clinical information is available for all patients (14 female and 37 male) and the mean age of these patients was 57.12 years [range: 26–73 years; stage: Ia to IIIa as per the 2004 World Health Organization criteria (Travis et al., 2006)]. Clinical information follow-up records are also available for the period until July 2013. TMA2 was used to analyze unpaired differential analysis of *SLC6A8* expression levels in tumor tissues of 51 patients and paraneoplastic tissues of 10 patients. Patients in both TMA1 and TMA2 had not received chemotherapy, radiotherapy, or biologic therapy before surgery.

### Immunohistochemical Staining and Quantification Analysis

To detect the expression of *SLC6A8* in LUAD, immunohistochemistry was performed using the standard indirect immunoperoxidase procedure. Paraffin specimens were cut into 4-µm thick slices, mounted on slides, baked, deparaffinized, and hydrated according to conventional methods. Two hundred milliliters of 3% H_2_O_2_ and 1 ml of NaN_3_ were used to inactivate the endogenous peroxidase activity, followed by antigen recovery performed with 10 mM sodium citrate buffer (pH 6.0). Slides were then incubated for 1 h at room temperature in 10 mM TBS with 4% normal rabbit serum (Proteintech, China) prior to incubation with primary antibody against *SLC6A8* (1:50, 20299-1-AP, Proteintech, China) at 4°C overnight. Then, the slides were developed in secondary antibody (1:200, K5007, DAKO, China) for 35 min at 37°C. Finally, the slides were weakly re-stained with hematoxylin at 37°C, dehydrated, and covered with coverslips.

To quantify the expression of *SLC6A8* protein in LUAD tissues, the slides were imaged with a microscope (Nikon Corporation; magnification). The images obtained were then converted to grayscale using ImageJ software (Schneider et al., 2012) by selecting “8-bit.” The “Uncalibrate OD” function was used to convert the grayscale values to optical density values. Using the “Set Measurement” module, we can set the area to be stained in the image. Next, the average optical density (AOD) was obtained by summing the optical density of the stained area over the stained area. Finally, paired difference analysis of AOD in tumor tissue and normal tissue in TMA1 was performed using GraphPad Prism software, while unpaired difference analysis was performed in TMA2.

### Genetic Alteration of *SLC6A8* in Lung Adenocarcinoma

Six datasets containing data from patients with LUAD (TCGA, Nature 2014; TCGA, Firehose Legacy; TCGA, PanCancer Atlas; MSKCC, Science 2015; Broad, Cell 2012; OncoSG, Nat Genet 2020) were selected to analyze the genetic alterations of *SLC6A8* with the cBioPortal database (http://www.cbioportal.org/). The samples in the selected datasets were divided into altered (*n* = 57) and unaltered groups (*n* = 1466) for prognostic analysis *via* the Kaplan-Meier survival analysis method.

### Prognostic Analysis of *SLC6A8* in Lung Adenocarcinoma

To validate the potential of *SLC6A8* as a survival biomarker, the Kaplan-Meier Plotter online database (https://kmplot.com/analysis/) was used to conduct survival analysis in the LUAD datasets. Based on the median value of gene expression, we divided *SLC6A8* data (Affy ID: 202219_at) into a high-expression group and a low-expression group, and then plotted Overall Survival (OS) (*n* = 719; cut-off value = 203) and First Progression (FP) (*n* = 461; cut-off value = 196) curves using the database. In addition, GraphPad Prism was used to classify samples from TMA2 into high- and low-expression groups based on the median AOD value (0.760) and to compare the survival significance between the two groups. Finally, using the “multivariate Cox analysis” board in Kaplan-Meier Plotter online database, we searched for LUAD independent prognostic factors by taking *SLC6A8*, gender, smoking history, stage, and T-stage and N-stage with OS into analysis (*n* = 131). T-stage as “tumor size,” M-stage as “metastasis,” and N-stage as “nodes.”

### Analysis of the Tumor Immune Microenvironment in Lung Adenocarcinoma

Firstly, we employed the R package Cell type Identification by Estimating Relative Subsets of RNA Transcripts (CIBERSORT; https://cibersort.stanford.edu/). This is a method to qualify and quantify 22 types of immune cells in the tissue, to visualize immune cell infiltration in LUAD, and to analyze the mRNA expression matrix of *SLC6A8* (Newman et al., 2015) (cancer vs. normal = 511 vs. 58).

Next, the TISIDB (http://cis.hku.hk/TISIDB/index.php) website, an integrated repository portal for tumor-immune system interactions (Ru et al., 2019) (*p*-value <0.05), was used to analyze the correlation between *SLC6A8* expression and immune cells in pan-cancer. Estimation of stromal and immune cells in malignant tumor tissues using expression data (ESTIMATE) was performed by using transcriptional profiles of cancer samples to infer the content of tumor cells as well as infiltrating immune and stromal cells, and using the “estimateScore” function to calculate tumor purity, immune cell score, and stromal cell score for all samples (Yoshihara et al., 2013; Becht et al., 2016). Then, to analyze the correlation between *SLC6A8* expression and subtype typing, the immune subtypes in LUAD were divided into six categories and subject to the Kruskal–Wallis test. These categories were: C1 (wound healing, n = 83), C2 (IFN-gammadominan, *n* = 147), C3 (inflammatory, *n* = 179), C4 (lymphocyte depleted, *n* = 20), C5 (immune-logically quiet, *n* = 0) and C6 (TGF-b dominant, n = 28).

Finally, the Pearson correlation analysis was used to explore the correlation between *SLC6A8* expression and a total of 64 immunomodulators, composed of immunoinhibitors and immunostimulators in LUAD in TCGA date. The Bonferroni-corrected threshold *p*-value was set at 0.0007 (0.05/64) considered statistically significant. The STRING website (https://www.string-db.org/online) was used to construct a network of protein interactions with the same set of immunomodulators associated with *SLC6A8* expression. Gene Ontology (GO) functional annotation and Kyoto Encyclopedia of Genes and Genomes (KEGG) pathway enrichment analysis was also performed on these immunomodulators using the WebGestalt online tool (http://www.webgestalt.org/).

### Construction of *SLC6A8*-Related Immune Signatures and Performance Evaluation

We used the Least Absolute Shrinkage and Selection Operator (LASSO) regression algorithm to select 44 immunomodulators associated with *SLC6A8* expression by 10-fold cross-validation of the “glmnet” and “survival” packages for penalty parameters. Then, the screened immunomodulators from LASSO were subjected to stepwise multivariate Cox proportional hazard regression analysis to obtain the optimal candidates and construct an immune-related risk model. The formula for calculating the risk score was as follows:

$$Risk\;score=\sum_{i=1}^{n}coefi\;x\;Xi\;$$

Where “coefi” and “Xi” represent the coefficient and expression level of each of the *SLC6A8*-related immunomodulators, respectively. In order to verify the accuracy of the model, the TCGA data are randomly divided into testing (n = 246) and training sets (*n* = 248), and the model was first constructed from the data in the training set, followed by the validation of the model in the testing set. Patients with LUAD were classified into high-risk and low-risk groups based on the median score as the risk cut-off point. The survival curves of the two groups were plotted according to the Kaplan-Meier method using the “survival” and “survminer” R packages in R. The “survivalROC” package was used to perform ROC curve, and the Area Under Curve (AUC) values were obtained to evaluate the prognostic model’s reliability.

### Construction of Nomogram Prognostic System

To further assess the practical clinical benefits of *SLC6A8*, the “rms” R package was used to build the nomogram scoring system. The nomogram scoring system develops scoring criteria based on the magnitude of the regression coefficients of all independent variables, giving each independent variable a score for each value level taken. A total score can then be calculated for each patient, and then these scores were used to assess the survival probability for each patient is calculated by a conversion function between the score and the probability of occurrence of the outcome (Becht et al., 2016). Finally, calibration curves were plotted to assess the accuracy of the model in predicting 1-, 3- and 5-years survival rates. If the model prediction curve coincides exactly with the reference line, the predicted value is equal to the actual value; if the model prediction curve is above the reference line, the predicted value is greater than the actual value; if the model prediction curve is below the reference line, the predicted value is lower than the actual value.

### Statistical Analyses

We implemented all statistical analyses with R (version 4.1.0) and GraphPad Prism 9 (version 9.1.2). For quantitative data in the article data analysis, statistical significance of normally distributed variables was estimated using Student’s t-test, and non-normally distributed variables were analyzed using the Wilcoxon rank-sum test. The log-rank test was used to compare data between two groups, and the Kruskal–Wallis test was performed to compare data between more than two groups. *p*-values <0.05 were considered statistically significant, whereas in the immunomodulator correlation analysis, the Bonferroni-corrected threshold *p*-value was set at 0.0007 (0.05/64) was considered statistically significant.

### Ethical Statement

This study was reviewed and approved by the Research Ethics Committee of The Affiliated Changzhou No. 2 People’s Hospital of Nanjing Medical University. All patients were properly briefed and voluntarily signed an informed consent form for the collection of clinical tissue samples. All specimens were processed and anonymized according to ethical and legal standards.

## Results

### Expression of *SLC6A8* in Lung Cancer

**FIGURE 1:**
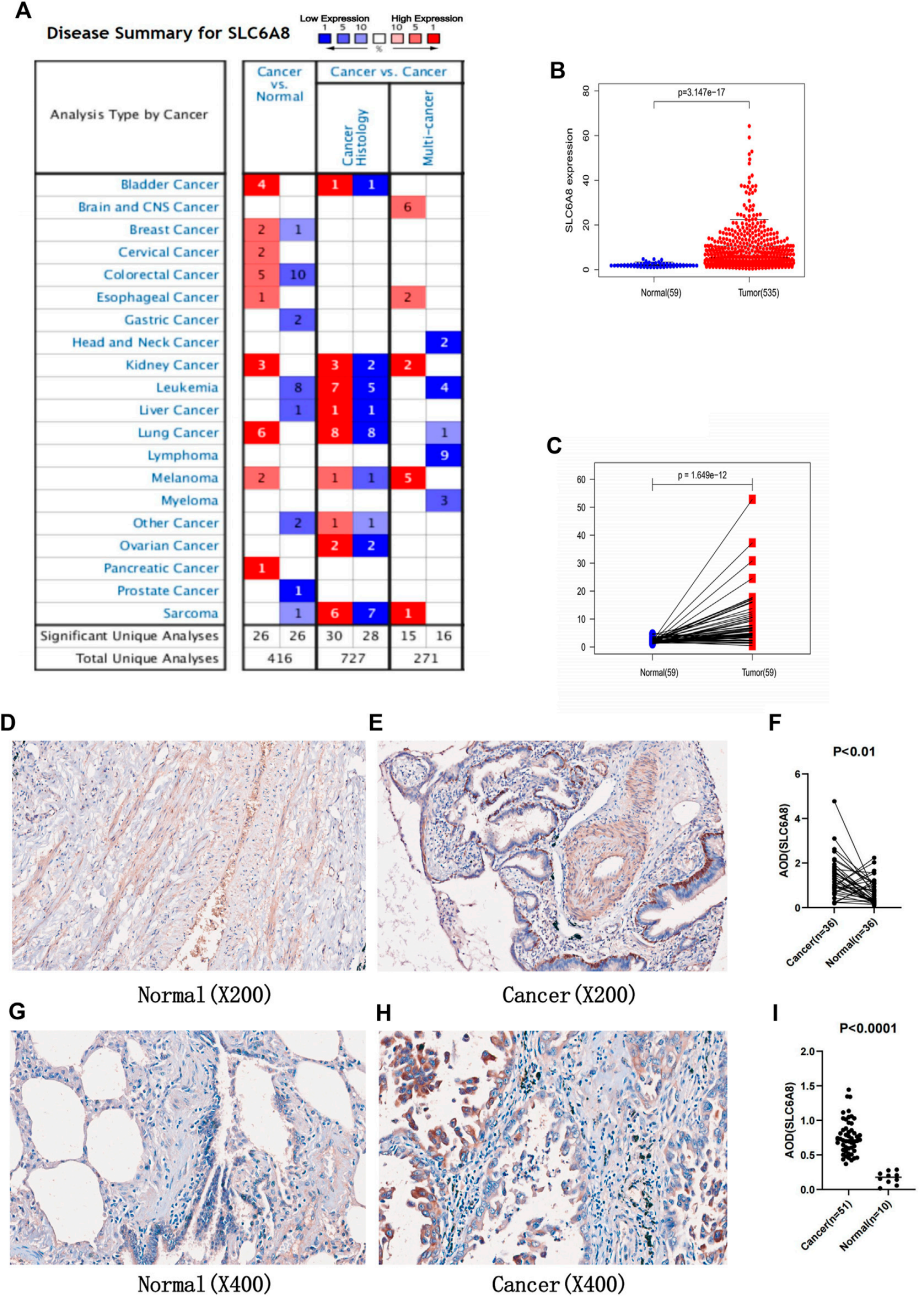
Expression of *SLC6A8* in tumors **(A)** Exprssion of *SLC6A8* in pan-cancer in the Oncomine database. Numbersrepresent the number of studies. Red represent high expression. while blue represents low exprssion **(B)** Unpaired differential analysis and **(C)** paired differential analysis of *SLC6A8* in LUAD in the TCGA database. **(F)** Analysis of paired differences between **(D)** normal and **(E)** LUAD tissues in TMA1 immunohistochemical staining. **(I)** Analysis of unpaired diffences between **(G)** normal and **(H)** LUAD tissues in TMA2 immunohistochemical staining.

Analysis from the Oncomine database revealed that *SLC6A8* was highly expressed in a variety of cancers in which lung cancer is one of them (Figure 1A). Among the subtypes of lung cancer, the expression of *SLC6A8* was higher in LUAD compared to normal tissue, according to the datasets of Stearman et al. (2005), Hou et al. (2010), Su et al. (2007). Based on the datasets of Bhattacharjee and Hou (Hou et al., 2010), *SLC6A8* was also overexpressed in lung squamous carcinoma. Moreover, *SLC6A8* was expressed at a high level in large cell lung cancer compared to normal tissue according to the dataset of Hou et al. (2010) (Table 1). Then, compared to normal tissues, it was demonstrated that *SLC6A8* was overexpressed in LUAD in both unpaired differential analysis (Figure 1B) and paired differential analysis (Figure 1C) using data from TCGA (*p*-value<0.05). Finally, paired difference analysis of TMA1 immunohistochemical pre-experiment results showed that *SLC6A8* was significantly higher in LUAD than in paraneoplastic tissue (*p*-value<0.05, Figures 1D–F). In addition, unpaired difference analysis in TMA2 exhibited the same results (*p*-value<0.05, Figures 1G–I). Altogether, *SLC6A8* expression is elevated in lung cancer and its subtypes compared to normal tissues.

**TABLE1:** The expression of *SLC6A8* in subtypes of lung cancer.

Types of lung cancer vs. Normal tissues	Fold change	*p* value	t-Test	References
Lung Adenocarcinoma vs. Normal	3.778	**6.69E-08**	6.854	Stearman et al. (2005)
Lung Adenocarcinoma vs. Normal	2.468	**2.78E-11**	8.213	Hou et al. (2010)
Lung Adenocarcinoma vs. Normal	2.401	**2.52E-06**	5.344	Su et al. (2007)
Squamous Cell Lung Carcinoma vs. Normal	85.234	**4.70E-10**	8.613	Bhattacharjee
Squamous Cell Lung Carcinoma vs. Normal	17.188	**7.59E-20**	19.869	Hou et al. (2010)
Large Cell Lung Carcinoma vs. Normal	2.856	**1.26E-05**	5.513	Hou et al. (2010)

Bold: *p*-value<0.05.

### Genetic Alterations and Prognostic Analysis of *SLC6A8* in Lung Adenocarcinoma

**FIGURE 2:**
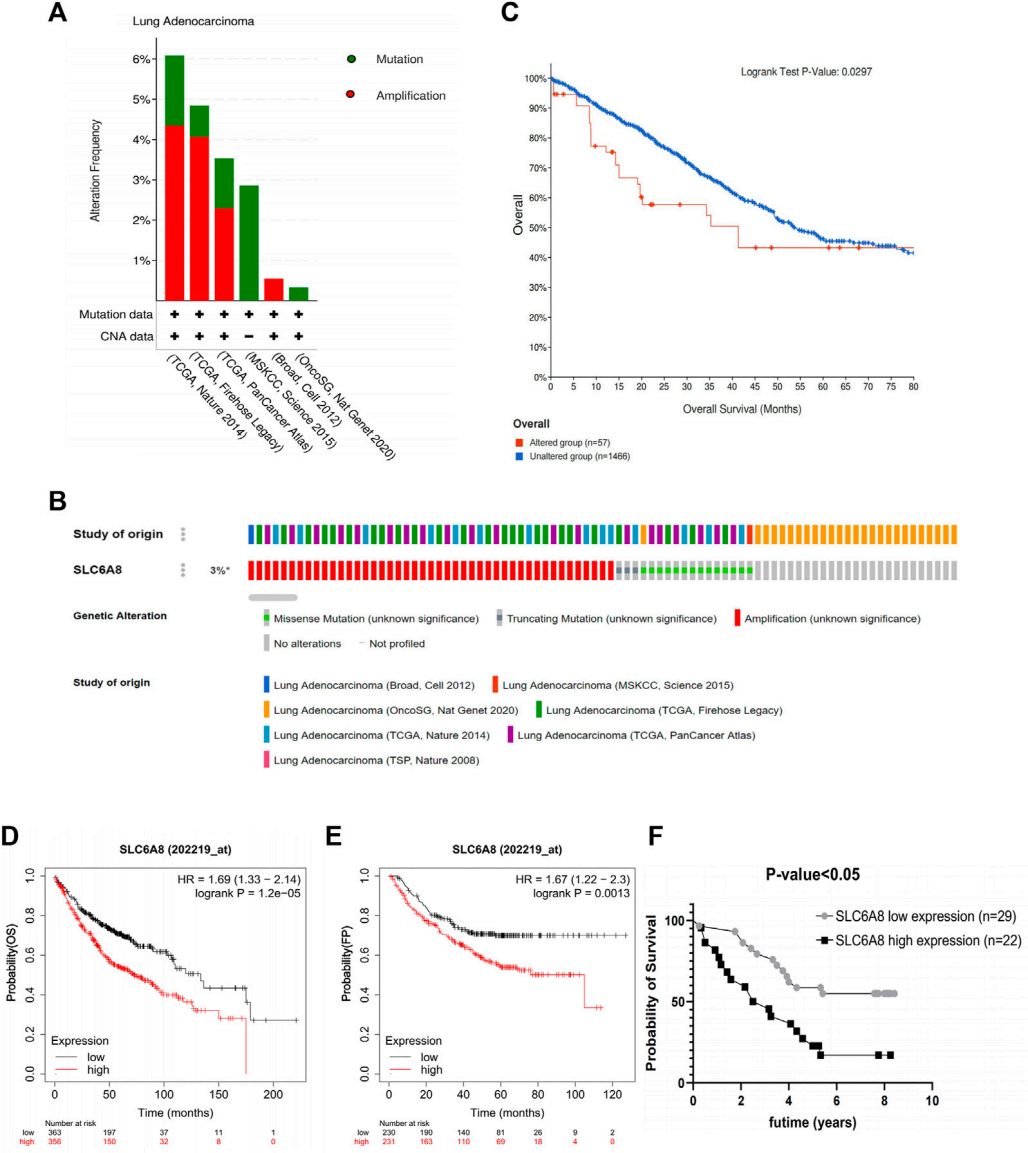
Genetic alteration and prognosis analysis of *SLC6A8* in LUAD. **(A**,**B)** Genetic alteration analysis of *SLC6A8 in* LUAD In the eBioportal database. **(C)** Prognostic analysis of *SLC6A8-*altered group and *SLC6A8-*unaltered group in LUAD. Correlation of *SLC6A8* in LUAD with **(D)** OS and **(E)** FP as per the results of the Kaplen_Meier plotter online website **(F)** Survival analysis of *SLC6A8* expression in TMA2 clinical follow up data information.

Gene alteration analysis revealed that 3% of the LUAD samples in the cBioPortal database were altered, mainly in the form of mutations and amplifications (Figures 2A,B). The samples in the database were divided into *SLC6A8*-altered and *SLC6A8*-unaltered groups, and the results indicated that the *SLC6A8*-altered group had a poorer prognosis (*p*-value<0.05, Figure 2C).

Then, the samples were divided according to *SLC6A8* expression into high- (OS: n = 356; FP: *n* = 231) and low- (OS: *n* = 363; FP: *n* = 230) expression groups according to the Kaplan-Meier Plotter survival analysis website. High *SLC6A8* expression in LUAD was found to be associated with poor OS and FP (*p*-value<0.05, Figures 2D,E).

Next, integrating TMA2 data with collected survival data and divided into high- and low- expression groups according to the median value of AOD of TMA2 (0.760). Similar to the previous results, the high-expression group was significantly associated with poorer prognosis compared to the low-expression group (*p*-value<0.05, Figure 2F). Moreover, multivariate Cox analysis indicated that *SLC6A8* and T-stage might be used as indicators of poor prognosis in LUAD independent of the clinical factors in table (*p*-value<0.05, Table 2). Therefore, we found that *SLC6A8* in LUAD is generally associated with poor prognosis.

**TABLE 2:** Multivariate cox analysis of *SLC6A8* and clinical factors in LUAD.

ID	HR	HR.95L	HR.95H	*p* value
SLC6A8	2.04	1.13	3.71	**0.0189**
Gender	1.72	0.94	3.16	0.0811
Smoking history	1.04	0.47	2.29	0.932
Stage	2.54	0.47	13.61	0.277
T	2.17	1.07	4.42	**0.0321**
N	1.36	0.25	7.37	0.723

Bold: p-value<0.05.

### Tumor Immune Microenvironment Analysis in Lung Adenocarcinoma

**FIGURE 3:**
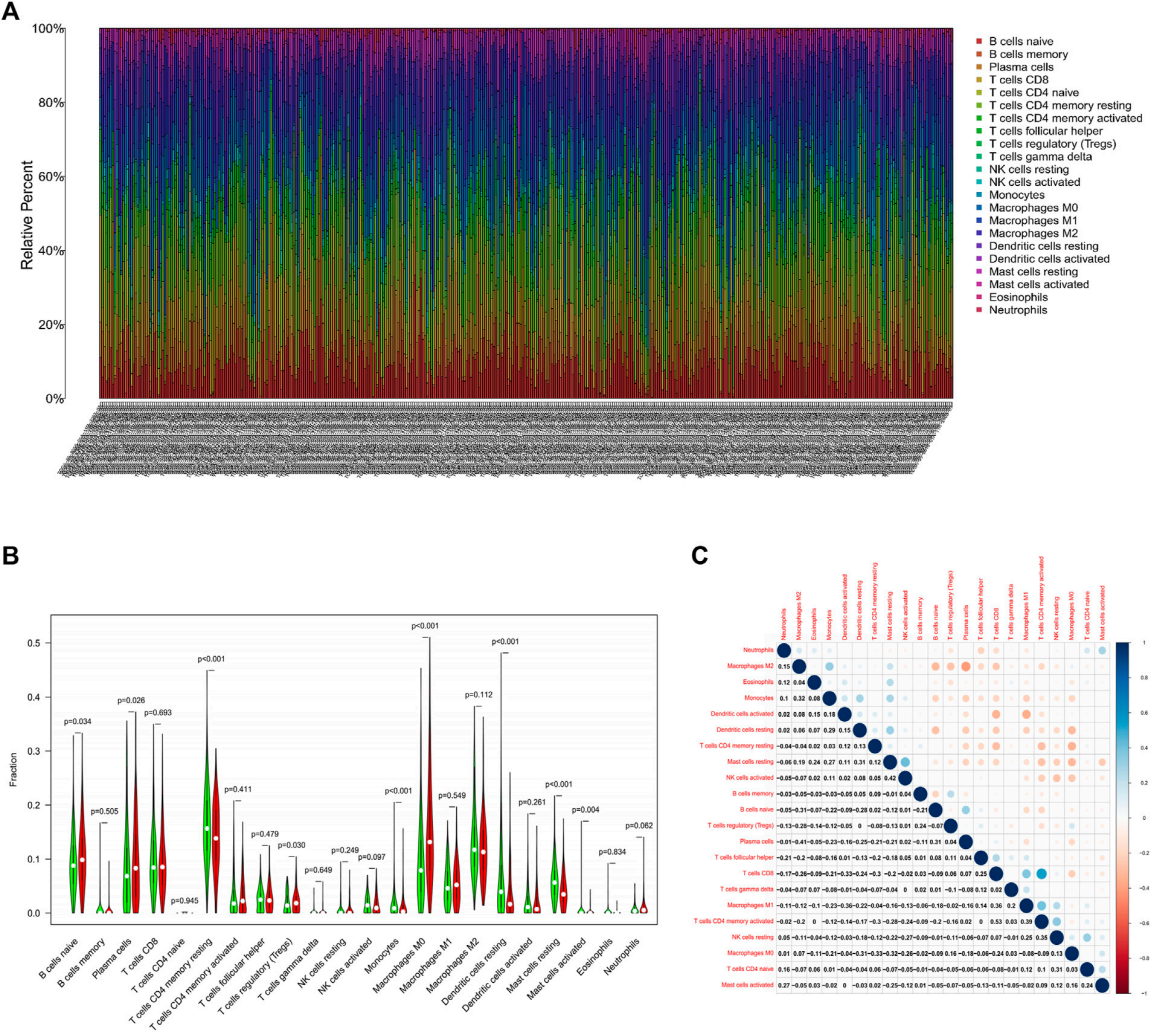
Analysis of the TIME in LUAD.**(A)** Visualization of immune cell infiltration in LUAD. X-axis represents the sample number of 535 patients in the TCGA database (Supplementary Material S1) **(B)** Differential analysis of infiltrated immune cells in LUAD tissues compared to normal tissues. **(C)** Correlation analysis of infiltrated immune cells iin LUAD.

Immune cell infiltration in LUAD was visualized using CIBERSORT (Figure 3A). Compared to normal tissue, the differential analysis of immune cell infiltration revealed that LUAD had increased infiltration of naïve B cells, plasma cells, regulatory T cells, and M0 macrophages. In contrast, there was decreased infiltration of CD4 memory and resting T cells, monocytes, resting dendritic cells, and resting and activated mast cells (Figure 3B). Correlation analysis among 22 immune cells identified that CD8 and activated CD4 memory T cells had the most significant positive correlation in LUAD, while the most significant negative correlation was found between plasma cells and M2 macrophages in LUAD (Figure 3C).

### Correlation Analysis of *SLC6A8* and the Tumor Immune Microenvironment in Lung Adenocarcinoma

**FIGURE 4:**
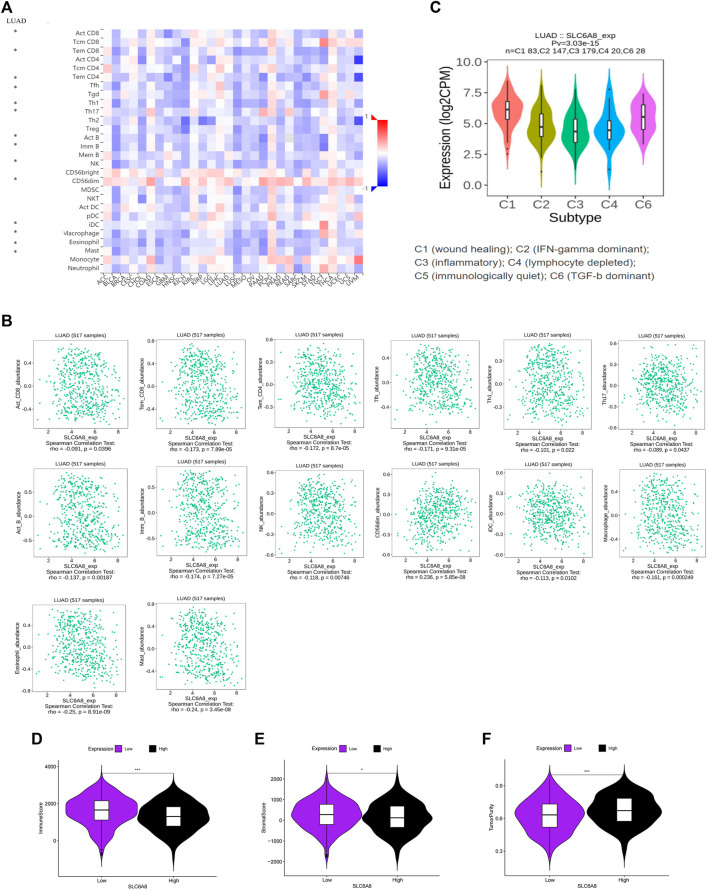
Association between *SLC6A8* expression with immune cells in LUAD **(A)** Correlation analysis of *SLC6A8* expression with cells in pan-cancer. **(B)** Correlation of *SLC6A8* expression with immune cells in LUCAD. **(C)** Association between *SLC6A8* expression and immune subtypes in LUAD. Calculated **(D)** immune cell score. **(E)** immune stroma score, and **(F)** tumor purity score in the LUAD immune microenvironment based on the ESTIMATE algorithm *:*p*-value<0.05; **:*p*-value<0.01; ***:*p*-value<0.001.

The TISIDB website analysis indicated that the immune cells associated with *SLC6A8* expression were correlated with multiple cancers (Figure 4A). Then we focused on LUAD specifically and found that Act_CD8, Tem_CD8, Tem_CD4, Tfh, Th1, Th17, Act_B, Imm_B, NK, CD56dim, iDC, Macrophage, Eosinophil and Mast were significant correlation with *SLC6A8* expression in LUAD, and most immune cells were negatively correlated with *SLC6A8* expression (*p*-value<0.05, Figure 4B). Assessment of the TIME of LUAD in TCGA using the ESTIMATE algorithm also presented consistent results, with the *SLC6A8* high-expression group showing significantly lower immune scores (Figure 4D) and stromal scores (Figure 4E) than the *SLC6A8* low-expression group, while the tumor purity scores (Figure 4F) exhibited an opposite phenomenon.

These results suggest that high *SLC6A8* expression was associated with less immune cell infiltration, which further supports the results of our previous survival analysis from the immunological aspect. Furthermore, the correlation between *SLC6A8* expression and immune cell subtypes (C1, C2, C3, C4, C6) was also found in LUAD, and further demonstrated that *SLC6A8* expression was lower in the immune-related subtypes (C2, C3, C4; *p*-value<0.05; Figure 4C). In conclusion, a close relationship between *SLC6A8* and the TIME in LUAD was found in this study.

### Immunomodulators Associated With *SLC6A8* Expression in Lung Adenocarcinoma

**FIGURE 5:**
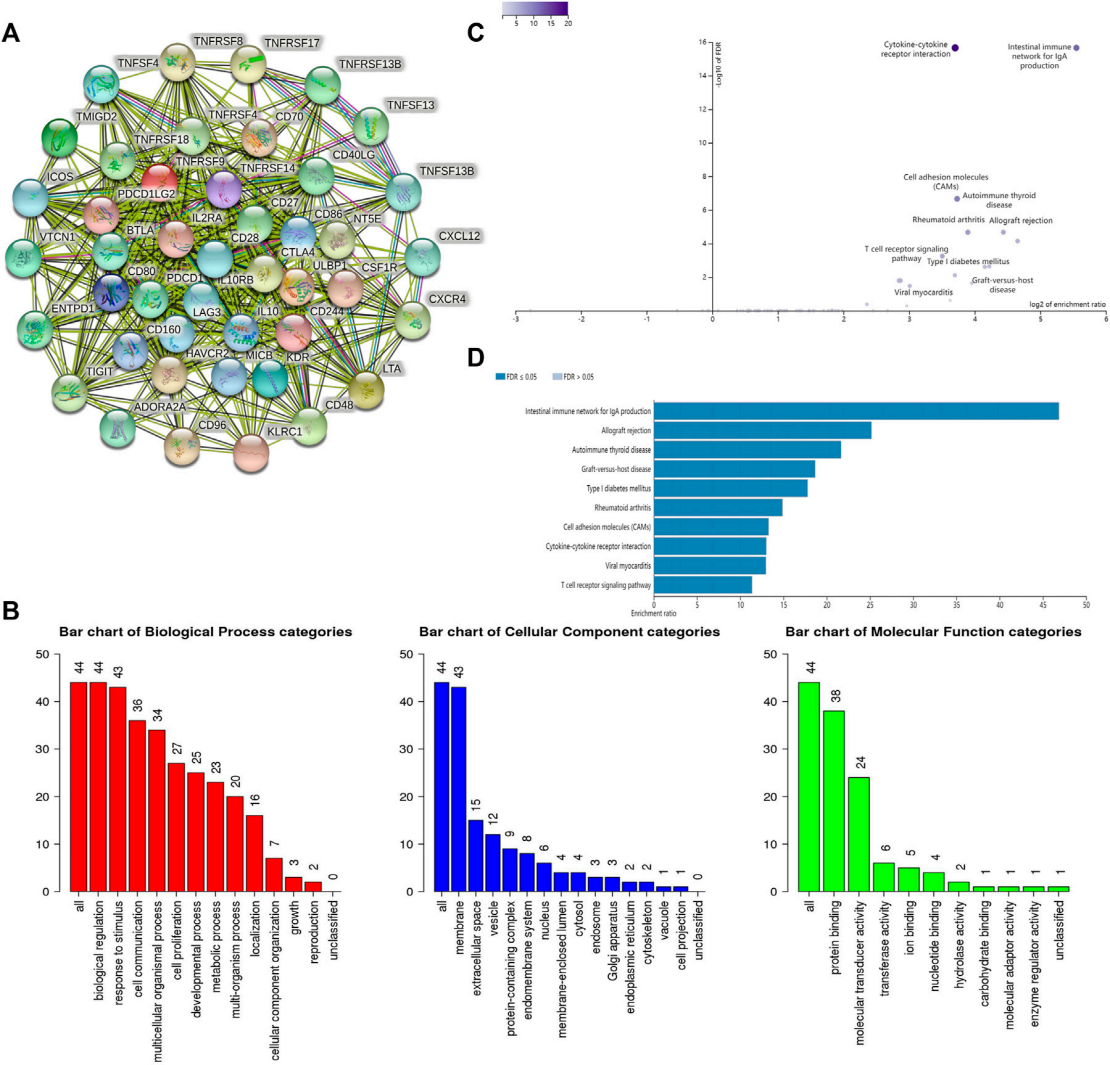
Analysis of SLC6A8-associated immunomodulators. **(A)** Protein interaction network of SLC6A8-associated immunomodulators inn LUAD as mapped using the STRING database.**(B)** GO and **(C**,**D)** KEGG analysis of SLC6A8- associated immunomodulators in LUAD using the WebGestalt website.

Pearson correlation analysis identifies 44 immunomodulators (*ADORA2A, BTLA, CD160, CD96, CSF1R, CTLA4, HAVCR2, IL10, IL10RB, KDR, LAG3, PDCD1, PDCD1LG2, TIGIT, VTCN1, CD27, CD28, CD40LG, CD48, CD70, CD80, CD86, CXCL12, CXCR4, ENTPD1, ICOS, IL2RA, KLRC1, LTA, MICB, NT5E, TMIGD2, TNFRSF13B, TNFRSF14, TNFRSF17, TNFRSF18, TNFRSF4, TNFRSF8, TNFRSF9, TNFSF13, TNFSF13B, TNFSF4, ULBP1, KIR2DL1*) in the TCGA database associated with *SLC6A8* expression in LUAD (Table 3). Next, we performed the protein interaction network analysis of these 44 immunomodulators using the STRING website (https://cn.string-db.org/) and found strong interactions between these immunomodulators (Figure 5A). GO analysis revealed 44 *SLC6A8*-associated immunomodulators in biological processes mainly enriched in biological regulation and response to stimulation. In terms of cellular components, mainly located in the cell membrane and molecular functional aspects are mainly protein binding (Figure 5B). KEGG enrichment analysis indicated that these immunomodulators are mainly involved in the immune regulation of the body, such as intestinal immune network for lgA production, allograft rejection, T cell receptor signaling pathway, ect (Figures 5C,D). This therefore implies that *SLC6A8* plays an essential role in the TIME of LUAD.

**TABLE 3:** The correlations between expression of *SLC6A8* and immunomodulators in LUAD.

immuneCell	Gene	cor	Correlation analysis (p-value)	Bonferroni correction (p-value)
B cell	ADORA2A	-0.2186	7.61E-07	**4.87E-05**
B cell	BTLA	-0.2661	1.62E-09	**1.04E-07**
CD8^+^ T cell	CD160	-0.245	3.00E-08	**1.92E-06**
CD8^+^ T cell	CD244	-0.343	3.39E-15	**2.17E-13**
CD4^+^ T cell	CD274	0.0448	0.316	1
M1 macrophage	CD96	-0.3213	2.16E-13	**1.38E-11**
M1 macrophage	CSF1R	-0.4045	0	**0**
M1 macrophage	CTLA4	-0.3355	1.52E-14	**9.71E-13**
M2 macrophage	HAVCR2	-0.4296	0	**0**
M2 macrophage	IL10	-0.3527	4.28E-16	**2.74E-14**
Neutrophil	IL10RB	-0.2	6.58E-06	**0.0004**
Neutrophil	KDR	-0.2669	1.44E-09	**9.20E-08**
Neutrophil	LAG3	-0.2835	1.20E-10	**7.69E-09**
Dendritic cell	LGALS9	-0.1786	5.88E-05	0.0038
Dendritic cell	PDCD1	-0.3456	1.99E-15	**1.27E-13**
Dendritic cell	PDCD1LG2	-0.2099	2.20E-06	**0.0001**
Dendritic cell	TGFB1	-0.0488	0.2746	1
Dendritic cell	TGFBR1	-0.0772	0.084	1
Dendritic cell	TIGIT	-0.2836	1.17E-10	**7.48E-09**
Dendritic cell	VTCN1	0.2222	4.95E-07	**3.17E-05**
Dendritic cell	CD27	-0.2916	3.31E-11	**2.12E-09**
Dendritic cell	CD28	-0.3363	1.30E-14	**8.30E-13**
Dendritic cell	CD40	-0.1614	0.0003	0.0184
Dendritic cell	CD40LG	-0.3385	6.37E-15	**4.08E-13**
Dendritic cell	CD48	-0.3787	0	**0**
Dendritic cell	CD70	-0.3348	1.74E-14	**1.12E-12**
Dendritic cell	CD80	-0.4041	0	**0**
Dendritic cell	CD86	-0.4133	0	**0**
Dendritic cell	CXCL12	-0.2908	3.81E-11	**2.44E-09**
Dendritic cell	CXCR4	-0.3742	0	**0**
Dendritic cell	ENTPD1	-0.3773	0	**0**
Dendritic cell	HHLA2	0.1645	0.0002	0.0137
Dendritic cell	ICOS	-0.3563	1.80E-16	**1.15E-14**
Dendritic cell	ICOSLG	-0.0529	0.2366	1
Dendritic cell	IL2RA	-0.3433	3.21E-15	**2.05E-13**
Dendritic cell	IL6	-0.0414	0.3547	1
Dendritic cell	IL6R	-0.012	0.788	1
Dendritic cell	KLRC1	-0.3372	8.18E-15	**5.23E-13**
Dendritic cell	KLRK1	-0.0407	0.3631	1
Dendritic cell	LTA	-0.307	2.65E-12	**1.70E-10**
Dendritic cell	MICB	-0.2433	3.75E-08	**2.40E-06**
Dendritic cell	NT5E	-0.2803	1.96E-10	**1.25E-08**
Dendritic cell	PVR	-0.0847	0.0581	1
Dendritic cell	RAET1E	-0.1475	0.0009	0.0594
Dendritic cell	TMIGD2	-0.3369	8.64E-15	**5.53E-13**
Dendritic cell	TNFRSF13B	-0.2052	3.56E-06	**0.0002**
Dendritic cell	TNFRSF13C	0.1105	0.0133	0.8497
Dendritic cell	TNFRSF14	-0.3035	4.77E-12	**3.05E-10**
Dendritic cell	TNFRSF17	-0.2263	3.00E-07	**1.92E-05**
Dendritic cell	TNFRSF18	0.2523	1.12E-08	**7.16E-07**
Dendritic cell	TNFRSF25	-0.0153	0.7326	1
Dendritic cell	TNFRSF4	-0.3045	4.00E-12	**2.56E-10**
Dendritic cell	TNFRSF8	-0.3153	6.28E-13	**4.02E-11**
Dendritic cell	TNFRSF9	-0.3649	1.49E-17	**9.53E-16**
Dendritic cell	TNFSF13	-0.2588	4.56E-09	**2.92E-07**
Dendritic cell	TNFSF13B	-0.454	0	**0**
Dendritic cell	TNFSF14	-0.194	1.24E-05	0.0008
Dendritic cell	TNFSF15	-0.1702	0.0001	0.0083
Dendritic cell	TNFSF18	0.1241	0.0054	0.3445
Dendritic cell	TNFSF4	-0.3246	1.20E-13	**7.67E-12**
Dendritic cell	TNFSF9	0.0325	0.4678	1
Dendritic cell	ULBP1	0.2295	2.18E-07	**1.39E-05**
Dendritic cell	KIR2DL1	-0.1464	0.001	0.0641
Dendritic cell	KIR2DL3	-0.1598	0.0003	0.0207

Bold: Bonferroni correction *p*-value<0.0007.

### Establishment of Immune Signatures Based on Three *SLC6A8*-Associated Immunomodulators and Predictive Evaluation

**FIGURE 6:**
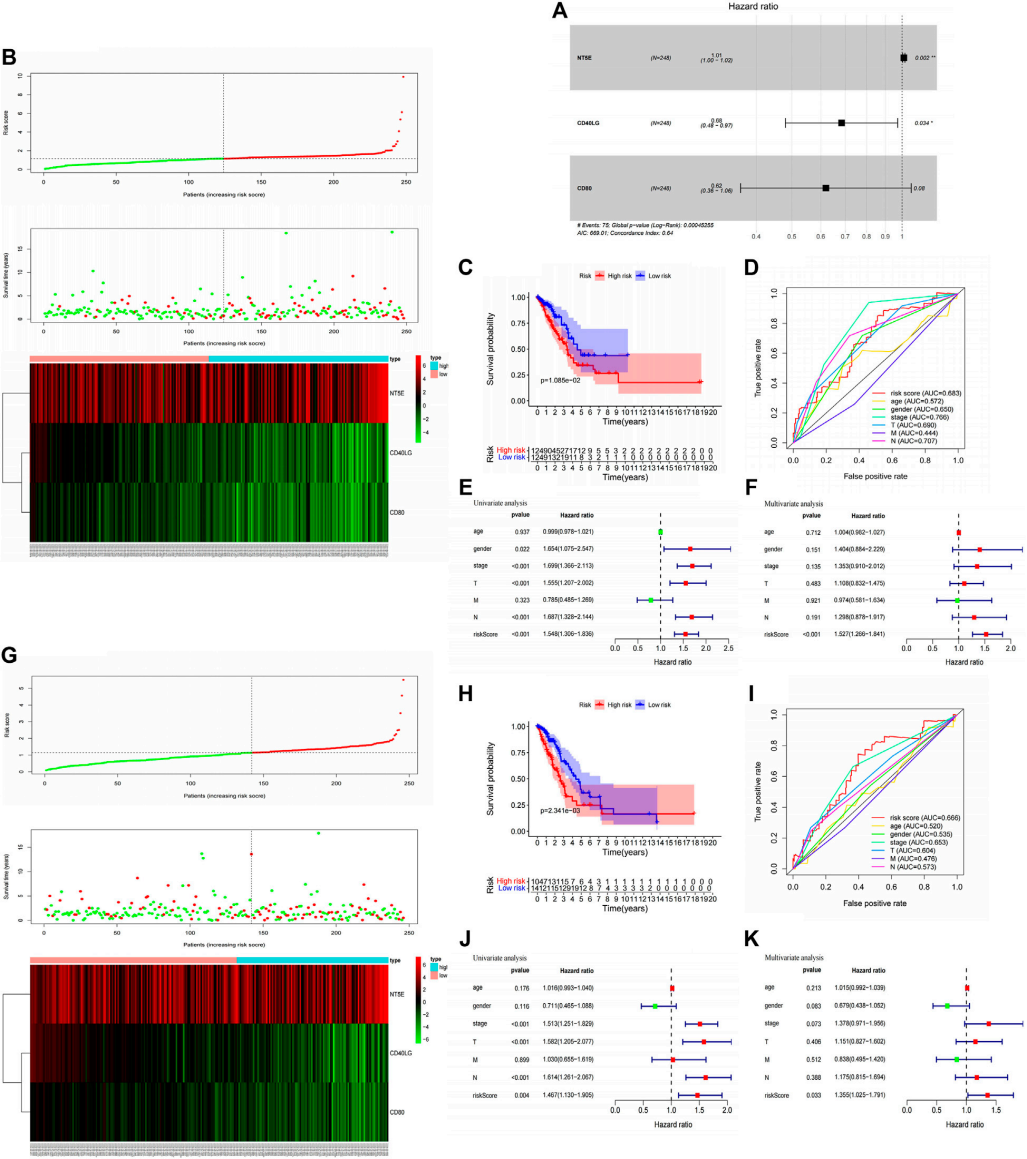
Construction immune signatures based on SLC6A8-associated immunomodulators and predictive evaluation. **(A)** Stepwise multivariate Cox Proportional hazard regression analyses to screen for three selected candidate immunomodulators (*NT5E, CD40LG, CD80*) to construct a set of immune signatures. Distribution of risk scores, survival statuses, and gene expression profiles for LUAD in **(B)** the training set and **(G)** testing set The *x*-axis of the heatmap represents the sample number of patients in the training set (Supplementary Material S2) and testing set (Supplementary Material S3) in the Cox risk proportional regression mode. Comparison of high - and low-risk groups based on model scoring using Kaplan-Meier survival analysis in **(C)** the training set and **(H)** testing set. Assesment of Cox risk regression model accuracy using ROC curves in **(D)** the training set and **(I)** testing set. Univariate and multivariate Cox analysis combining model scoring with clinical factors to identify independent prognostic indicators in **(E,F)** the training set and **(J)**, **(K)** testing set.

Immunomodulators are an important component of the tumor immune microenvironment, and their alterations are closely related to patient prognosis (Mahoney et al., 2015). Based on the list of44 immunomodulators associated with *SLC6A8* expression in the TIME of LUAD, we used LASSO regression and stepwise multivariate Cox proportional hazard regression analyses to screen for five candidate immunomodulators (*NT5E, CD40LG, CD80*) to construct a set of immune signatures (Figure 6A). There were the following computational formulas of the multivariate Cox proportional hazard model: risk core = (coefficient *NT5E* × Expression *NT5E*) + (coefficient *CD40LG* × Expression *CD40LG*) + (coefficient *CD80* × Expression *CD80*).

The training set was divided into high- and low-risk groups based on the median value (1.151) of the risk scores calculated by the model. We found that patients with LUAD in the low-risk group had better survival outcomes than those in the high-risk group (*p*-value<0.05, Figures 6B,C). The AUC of the ROC curve for the multivariate Cox proportional hazard model is 0.683, indicating amoderate accuracy of the model’s risk prediction capability (Figure 6D). Finally, univariate and multivariate Cox analysis of the proportional hazard model’s risk scoring combined with clinical factors such as age, gender, and grade suggested that the model’s risk score could be used as a risk factor independent of these clinical factors (*p*-value<0.05, Figures 6E,F). In addition, we further validated the findings in the training set with the testing set analysis. Based on the median values (1.147) of the model risk scores, we can well classify the patient sample in the testing set into high- and low-risk groups, and the high-risk group survives significantly worse than the low-risk group (Figures 6G,H). The predictive ability of the testing set in the ROC curve evaluation revealed an AUC value of 0.666, which also predicts amoderate accuracy (Figure 6I). Moreover, univariate and multifactor Cox analyses showed that model risk scoring in the testing set could be used as an independent prognostic risk factor (Figures 6J,K). The model analysis thus shows that *SLC6A8*-related immune signatures are associated with poor prognosis, and the immune signatures have good survival predictive efficacy.

### Constructing Nomogram Survival Prediction Program

**FIGURE 7:**
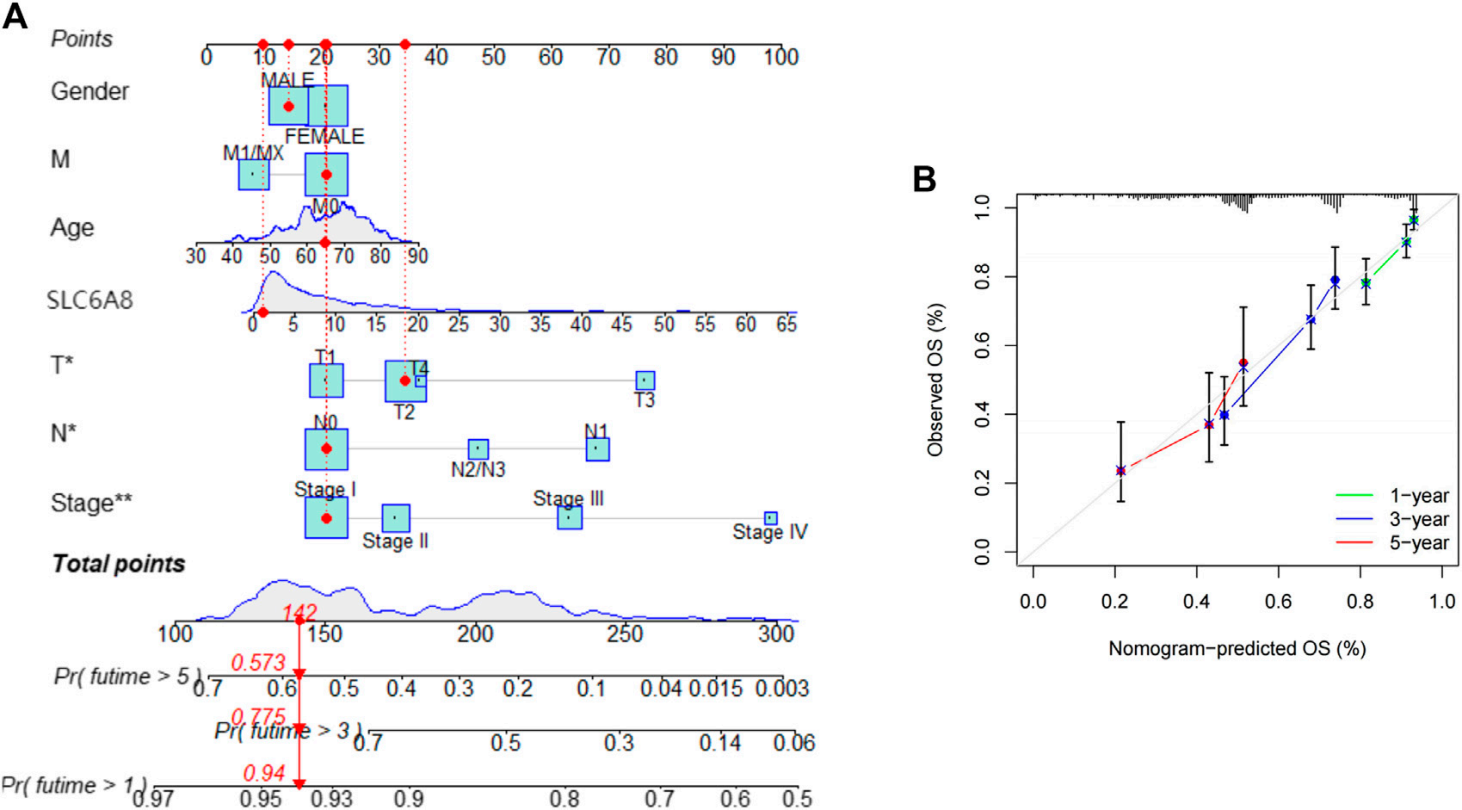
Assessing the practical clinical benifits of *SLC6A8* **(A)** Construction of a nomogram survival prediction system including *SLC6A8* expression and clinical factors. **(B)** Plotting calibration curves for nomogram 1-, 2- and 5- year survival prediction curves.

To assess the practical clinical benefit of *SLC6A8*, we combined *SLC6A8* expression with clinical factors (gender, age, stage, T-stage, N-stage and M-stage) to construct the nomogram survival prediction system to predict patient survival at 1-, 3-, and 5-year periods (Figure 7A). The red reference line in the nomogram indicates the best prognosis for the patient. In addition, we further plotted the calibration curves of the survival prediction curves and the results showed that the survival prediction curves for patients at 1-, 3-, and 5- years fluctuated slightly above and below the calibration curves, implying that the model’s predicted survival with a high degree of accuracy (Figure 7B).

## Discussion

Growing cancer incidence and mortality worldwide demands development of accurate biomarkers to perfect detection, diagnosis, prognostication, and monitoring (Costa-Pinheiro et al., 2015). Biomarkers of tumor diagnosis and prognosis are consistently a key area of interest for researchers. A wide range of biomarkers are now being proposed because they have shown superior biological benefits, such as cancer-derived exosomes (Kok and Yu, 2020) and extracellular vesicles (Urabe et al., 2020).

*SLC6A8* has been reported to be closely associated with the development of cancer. In NSCLC, *SLC6A8* may be involved in tumor progression through the Notch signaling pathway (Feng et al., 2021). In this study, we demonstrated that *SLC6A8* was overexpressed in LUAD *via* open databases and immunohistochemistry. Survival analysis of data from the Kaplan-Meier Plotter database and clinical follow-up showed that the *SLC6A8* high-expression group was associated with poor prognosis. Genetic alterations in *SLC6A8* were also shown to be linked to worse prognosis. Moreover, multivariate Cox regression analysis indicated that *SLC6A8* could be an independent risk prognostic factor for LUAD. Therefore, we consider that *SLC6A8* may serve as a prognostic biomarker for LUAD.

The TIME comprises tumor cells, immune cells, mesenchymal cells, and extracellular components. Studies have demonstrated that these components play a crucial role in the effectiveness of anti-tumor immunity at the cellular and tissue level and the degree of immune cell infiltration influence patient prognosis (Li et al., 2016; Binnewies et al., 2018). Hegde et al. first classified three immune phenotypes according to the different characteristics of the TIME, namely “immune infiltrative,” “immune rejection,” and “immune desert” (Hegde et al., 2016). Survival analysis revealed that the phenotype of immune inflammation, characterized by adaptive immune cell infiltration and immune activation, survived better than the immune evasion and immune desert phenotypes (Zhang et al., 2020; Chong et al., 2021). In this study, we identified from the TISIDB database that the majority of immune cells associated with *SLC6A8* expression in LUAD showed a significant negative correlation. We thus speculate that high expression of *SLC6A8* in LUAD inhibits immune cell infiltration. Evaluation of the LUAD TIME by the ESTIMATE algorithm revealed that the immune cell score was significantly lower in the *SLC6A8* high-expression group than in the SLC6A8 low-expression group, while the opposite phenomenon was observed in terms of tumor purity. In addition, the immune-related subtypes (C2, C3, and C4) in LUAD have lower expression of *SLC6A8* compared to other subtypes. This further supports our speculation that *SLC6A8* overexpression in LUAD TIME mediates poor immune prognosis.

Marker proteins, also called immunomodulators, on the surface of immune cells in the tumor microenvironment can regulate the TIME. These proteins are classified as immunostimulators and immunoinhibitors, and studies have shown that immunomodulators have a significant impact on patient prognosis (Mahoney et al., 2015). To take this into consideration as a part of our prognostic model, we constructed immune signatures based on *SLC6A8*-related 44 immunomodulators. According to the multivariate Cox proportional hazard model, it indicated that the groups deemed high-risk by the model were associated with poor prognosis, and the model has good prediction accuracy in the training and testing sets. Moreover, univariate and multivariate Cox analyses demonstrated that the model’s risk scoring was an independent risk factor for the prognosis of LUAD patients. This further demonstrates that *SLC6A8* is associated with poor prognosis in the TIME of LUAD.

However, as most of our data comes from online databases, the veracity and applicability of the results is limited by data available these open databases. Clinical validation of the model is still necessary. Subsequent studies on the immune mechanism of *SLC6A8* associated with LUAD would also be essential in proving its potential as a prognostic biomarker.

In summary, we performed Kaplan-Meier survival analysis with respect to *SLC6A8* expression, multivariate Cox analysis from combining clinical factors, Kaplan-Meier survival analysis in genetic alterations, and immune prognostic analysis based on the TIME all suggest that *SLC6A8* is associated with poor prognosis in LUAD. Therefore, *SLC6A8* may act as a biomarker for prognosis in LUAD.

## Data Availability

The original contributions presented in the study are included in the article/Supplementary Material, further inquiries can be directed to the corresponding author.

## Abbreviations

AOD, average optical density; AUC, areas under curve; C-index, concordance index; CIBERSORT, cell type identification by estimating relative subsets of RNA transcripts; DFS, disease-free survival; ESTIMATE, estimation of stromal and immune cells in malignant tumor tissues using expression data; FP, first progression; LUAD, lung adenocarcinoma; GO, Gene Ontology; KEGG, kyoto encyclopedia of genes and genomes; LASSO, least absolute shrinkage and selection operator; TCGA, the cancer genome atlas; OS, overall survival; TMA, tissue microarray.
